# Effects of heavy metals on health risk and characteristic in surrounding atmosphere of tire manufacturing plant, Taiwan

**DOI:** 10.1039/c7ra10994f

**Published:** 2018-01-15

**Authors:** Chia-Hsiang Lai, Chia-Hua Lin, Chang-Chun Liao, Kuen-Yuan Chuang, Yen-Ping Peng

**Affiliations:** Department of Safety Health and Environmental Engineering, Central Taiwan University of Science and Technology Taichung Taiwan chlai2@ctust.edu.tw +8864-22391647; Department of Biotechnology, National Formosa University Yunlin 63208 Taiwan; Institute of Safety Health and Environmental Engineering, Central Taiwan University of Science and Technology Taichung Taiwan; Department of Safety Health and Environmental Engineering, Central Taiwan University of Science and Technology Taichung Taiwan; Department of Environmental Science and Engineering, Tung Hai University Taichung Taiwan

## Abstract

The health and environmental effects of metal-containing carbon black (CB) particles emitted from a CB feeding area near a tire manufacturing plant were investigated. The mass ratios of PM_1_ and PM_0.1_ (UFPs) relative to TSP were 13.84% ± 4.88% and 50.84% ± 4.29%, respectively. The most abundant elements in all fractions were Fe, Al, and Zn. The mean percentage contributions of Al, Fe, Zn, Cu, and Co to the coarse particles ranged from 49.1% to 69.1%, thus indicating that the Al, Fe, and Zn contents in the CB particles were affected by workplace emissions. The ratios of the total mean deposition fluxes of atmospheric particle-bound heavy metals in the human respiratory tracts of workers/adults, workers/children, and adults/children were approximately 5.5, 11.0, and 2.0, respectively. The integrated risks of five elements *via* two exposure pathways to adults and children were 1.1 × 10^−4^ and 1.7 × 10^−5^, respectively; these numbers reflect the high cumulative carcinogenic risk posed by these toxic metals to local residents (both adults and children; limit, 10^−6^). These results demonstrate the potential health risk presented by particle-bound heavy metals to humans residing near tire manufacturing plants *via* inhalation and dermal contact exposure.

## Abstract 2

The health and environmental effects of metal-containing carbon black (CB) particles emitted from a CB feeding area near a tire manufacturing plant were investigated.

## Graphical abstract

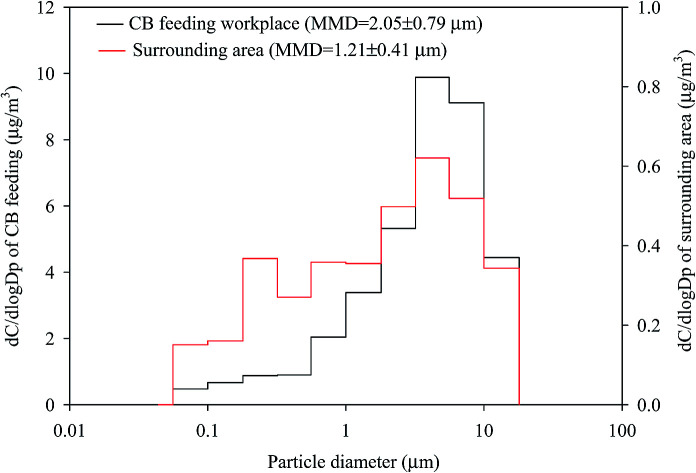

## Introduction

Carbon black (CB) is a manufactured product consisting of a fine powder of pure elemental carbon, organic compounds, and inorganics.^1–3^ CB is widely used in a number of common consumer products and industrial manufacturing (*e.g.*, rubber, ink, printing, paint, paper, plastics, ceramics, batteries, carbon electrode products, and tires) and has been studied intensively by health scientists in recent decades. Several studies focusing on CB exposure have addressed CB manufacturing and its production, collection, and handling.^4–7^ While epidemiological studies among workers in CB production and the rubber industry have provided inadequate evidence of carcinogenicity, overall data from cancer studies in rodents exposed to CB have gathered sufficient evidence of carcinogenicity. In fact, the Working Group has evaluated CB as a possible Group 2B carcinogen to humans.^4^

CB is poorly soluble and nano-sized.^4^ The increased biological potency of ultrafine particles is related to the content of redox cycling organic chemicals and their ability to damage mitochondria.^8^ Besides increasing reactive oxygen species, nano-sized CB causes loss of cell viability in endothelial cells and promotes adhesion of monocytes, both of which accelerate the development of atherosclerosis.^9^ CB aggregates cause endothelial dysfunction by activating ROCK.^10,11^ Moreover, trace elements (Ca, Cu, Fe, Mn, K, Pb, As, Cr, Se, and Zn) have been found in samples of CB.^12^ Cd, Cr and Pb are known to cause deleterious effects on human health.^13^ Arsenic (As) can cause serious disturbances of cardiovascular and central nervous system.^14^ Long-term exposure to Pb may lead to memory deterioration, prolonged reaction times and reduced ability to understand. Dietary Cr intakes were assumed to be directly correlated with breast cancer mortalities.^15^ Although CB is known to typically contain large quantities of elemental carbon and PAHs, some toxic components of the material, *e.g.*, metals, are not widely discussed. CB is emitted during industrial manufacturing, and the particles are deposited in the human respiratory tract by breathing and exposure to polluted environments. As the specific effects of the size distributions of metal-containing CB particles on workers and residents in tire manufacturing plants and the surrounding areas are not known, analyzing the hazards and risks of exposure to different sizes of metal-containing particles is an important and novel research topic.

The objective of this study is to collect and analyze size-segregated suspended particle samples for the presence of heavy metals. Respiration and dermal contact are regarded as significant intake pathways of human exposure to heavy metals. The CB feeding and surrounding areas of a tire manufacturing plant were selected as sampling sites, and the size distributions of heavy metals were characterized and used to estimate deposition fluxes in the human respiratory system *via* a model developed by the International Commission on Radiological Protection (ICRP). Finally, the carcinogenic effects of exposure to heavy metal-containing particles through inhalation and dermal contact were evaluated.

## Methodology

### Sampling site and sample collection

In Taiwan, many factories were built near the farmlands in the rural county more than 30 years ago. With the rapid development of rural economy and population, the adverse effects of pollutants on health and environment from the emitting sources raised severe public concern. The tire manufacturing plant was located in a rural county in Central Taiwan. Although several farmlands, irrigation ditches, and residential areas are present around this tire plant, no large industrial area is present. This study analyzed the ambient atmosphere of the CB feeding and surrounding areas at the tire manufacturing plant. Details of the tire production processes in the plant were described in our previous study.^16^ Four grades of CB products (*i.e.*, N-220, N-330, N-339, and N-660) are used in different tire products (*e.g.*, truck tires and vehicle tires).

**Fig. 1:**
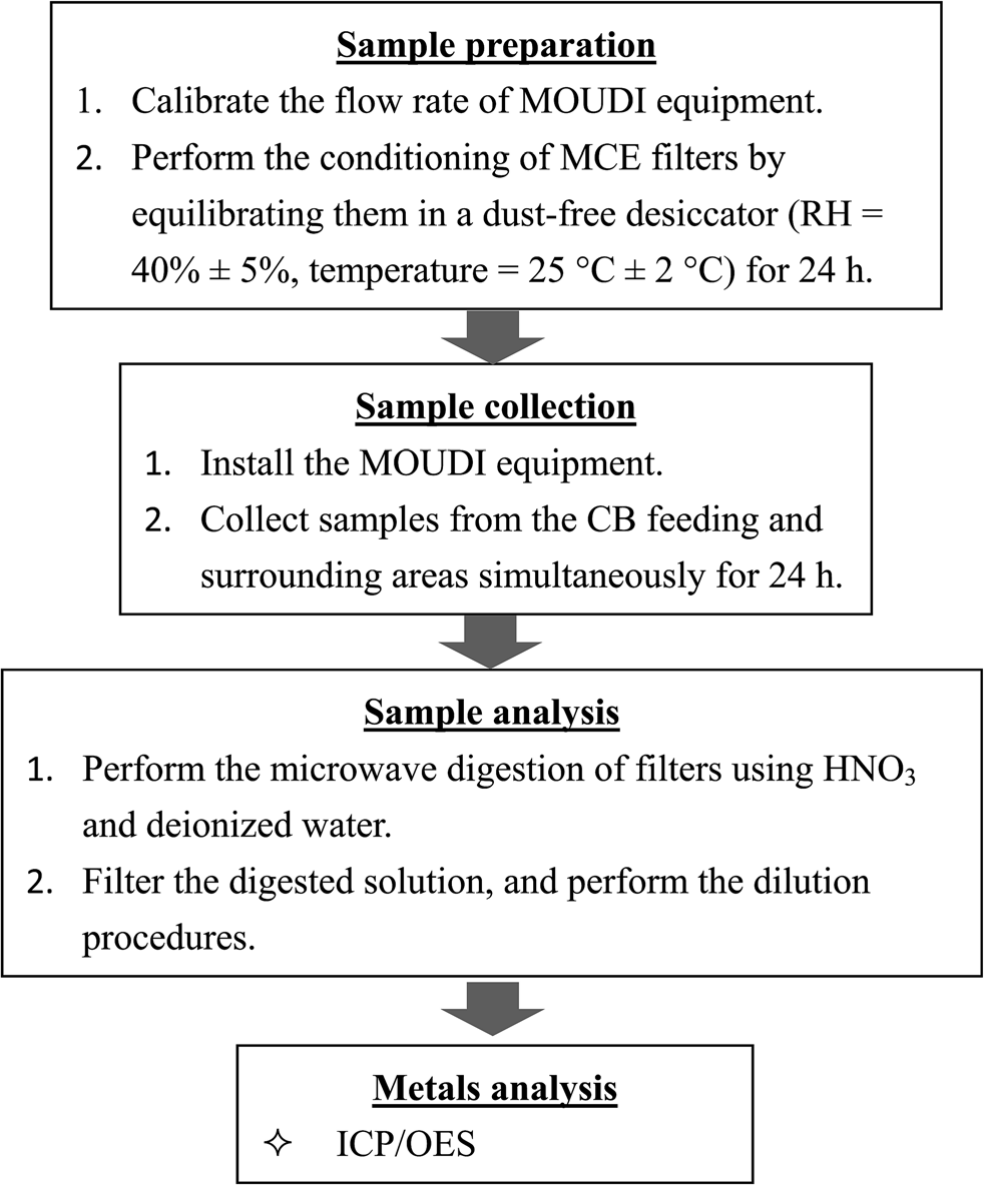
Flowchart for sampling and analysis of samples.

In this study, the sampled particles were collected from the feeding and surrounding area simultaneously during the feeding process. Atmospheric particles were collected at two sampling sites for 20 days in March and April 2016 using a 12-stage micro-orifice uniform deposit impactor (MOUDI II, model M110-R). Sampling was carried out at a flow rate of 30 L min^−1^ and air particulate samples of different size fractions were collected from each sampling site over 24 h. Four hundred and eighty air samples were collected. The Central Taiwan's weather station located at approximately 1.5 km toward north–northwest (NNW) of the sampling site provides meteorological parameters including pressure, temperature, wind speed, wind direction, and relative humidity (RH). The mean pressure was 1012.44 ± 2.57 (hPa), mean temperature was 22.34 ± 3.40 °C, mean RH was 83.62 ± 7.31%, and prevailing wind direction was northwest (NW) during the sampling periods. The sampling site was located downwind of CB-emitting source in surrounding area. The 50% cut off diameters (*D*_50_) of the MOUDI stages were 10.0, 5.6, 3.2, 1.8, 1.0, 0.56, 0.32, 0.18, 0.10, and 0.056 μm and included inlet (18 μm) and exit (<0.056 μm) stages. Size-segregated particles were classified as coarse (aerodynamic diameter > 1.8 μm) and ultrafine (aerodynamic diameter < 0.1 μm). The distance between the sampling site and the CB feeding area was approximately 2 m. Another sampling site representing the surrounding area was located near a fence about 15 m from the factory building. Several farmlands and irrigation rivers of agricultural land are affected by CB emissions near the tire manufacturing plant. The inlet of the MOUDI sampler place in the CB feeding area was set 1.5 m high from the ground. Ambient air samples of the surrounding area were also collected from the roof of a building located near the factory fence at a height of about 3 m above ground level. A total of 240 particle samples (20 samples containing 12 size fractions) were collected from every sampling site. Mixed cellulose ester (MCE) membrane filters (Advantec MFS Inc., 47 mm diameter, 0.45 μm pore size) were used as the substrate in all stages of collection the MOUDI sampler. Both blank and loaded MCE substrates were conditioned for 24 h by maintaining a humidity of 40% ± 5% and temperature of 25 ± 2 °C to equilibrate before and after each sampling. A laboratory standard weight of 10 mg was measured before, during, and after each weighing session to ensure accuracy of the weight measurements. Each filter was weighed on an electrical balance with a sensitivity of 10 μg, and the suspended particulate matter (PM) concentration was determined by dividing the particle mass by the volume of the sampled air. Fig. 1 shows a flowchart of the sampling and analysis procedures.

### Instrumental analysis and quality control

The concentrations of metal elements (Al, Cd, Co, Cr, Cu, Fe, Ga, In, Mn, Ni, Pb, Sr, and Zn) were analyzed by inductively coupled plasma-optical emission spectrometry (ICP-OES) (PerkinElmer Optima 8000 device). All samples collected by the MOUDI instrument were analyzed according to Method 7302 of the National Institute for Occupational Safety and Health. The loaded filters were then digested in HNO_3_*via* a microwave digestion system (Model: ETHOS One; Milestone Inc.) as follows:

Step 1: Each filter was placed in a 100 mL Teflon microwave digestion vessel with 10 mL of a mixture of concentrated HNO_3_ (70%, trace metal grade) and de-ionized water at a volume ratio of 1 : 1 in preparation for microwave digestion.

Step 2: The microwave temperature was increased from room temperature to 200 °C over 6.5 min.

Step 3: The temperature was maintained at 200 °C for another 10 min.

Step 4: The samples were cooled for 20 min, and the digested solution was diluted to 20 mL using 20% HNO_3_ in de-ionized water. The resulting diluted solution was used for metal analysis.

Calibration was performed using multi-element (metal) standards (*i.e.*, certified reference materials) in a 20% (v/v) HNO_3_ solution. The detection limit of the ICP-OES instrument for the 13 metals was 0.91–3.52 ng m^−3^. Field and laboratory blank samples were subjected to the same analytical procedure as the samples that were regularly analyzed, and sample data were corrected by subtraction of the filter blanks. This study measured recoveries with one medium blank and two spiked medium blanks per 20 samples. Recovery efficiencies were determined by performing the same experimental procedures using solutions containing known metal concentrations as samples. The recovery values of the metals ranged from 91.1% to 98.5%.

### Deposition fraction and health-risk assessment

To estimate the deposition efficiency and flux of inhaled particles in the human respiratory tract, this study adopted the simplified equations from the ICRP (1994) model.^17^ The model calculates size-resolved metals deposition in three main regions of the human respiratory tract, *i.e.*, (1) head airway (HA), (2) tracheobronchial region (TB), and (3) alveolar region (AR). The deposition efficiencies of size-specific particles in the HA (DF_HA_*i*__), TB (DF_TB_*i*__), and AR (DF_AR_*i*__) can be estimated by the following equations:1
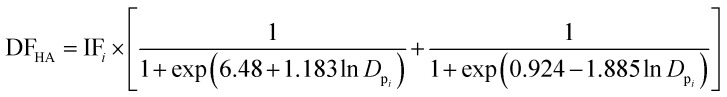
2
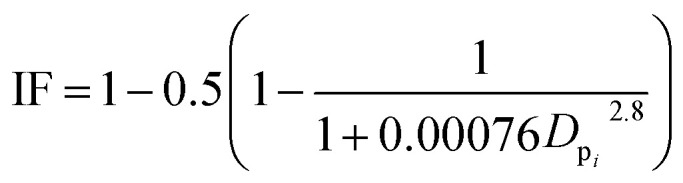
3
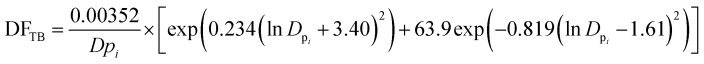
4
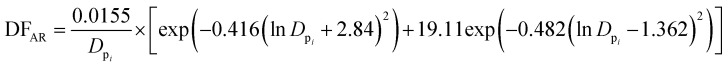

5 DF_T_ = ∑(DF_*i*_ × *C*_*i*_) × *V*

where *D*_p_*i*__ (μm) is the geometric mean diameter (GMD) of each size-segregated particle class (here, the GMD of size fraction >18 μm is defined as 20 μm (ref. 18)) and IF is the inhalable fraction of all particles; DF_T_ is the total deposition flux from inhalation (ng per day). The sum of the deposition efficiencies in all three regions was defined as the total deposition efficiency.

The health risk presented by metal inhalation to persons working in the CB feeding area was evaluated by quantifying the risk of developing cancer using a probabilistic approach. This study adopted the inhalation exposure model of the United States Environmental Protection Agency (US-EPA) baseline risk assessment approach.^19^ The EPA Integrated Risk Information Database and International Agency for Research on Cancer classify pollutants as carcinogens. This investigation found that Cr, Ni, Cd, and As are carcinogenic and could affect the human immune system through the respiratory system. The average daily exposure dose of metallic particles through inhalation (ADD_inh_) and dermal contact (ADD_derm_) were calculated using the following equations:^16,20^6
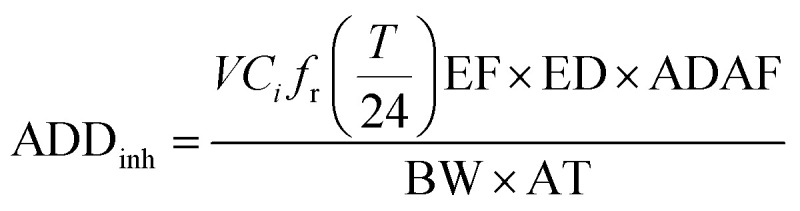
7
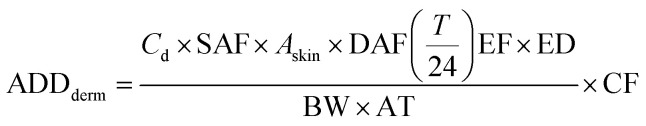


Cancer risk (CR) estimates were compared with carcinogenic regulatory levels of concern, *i.e.*, 10^−6^ to 10^−4^. Health risk can be quantified using the following equation:

8 CR = ADD × SF

where ADD denotes the life-averaged daily dose from inhalation and dermal contact (mg per day per kg); *C*_*i*_ denotes the air concentration of a metal (ng m^−3^), including the total concentration in all size fractions; *C*_d_ denotes the contact skin concentration of a metal (mg kg^−1^), including the total concentration in all size fractions; *f*_r_ is the deposition efficiency of particles in each region; *V* is the inhalation rate of humans, which was assumed to be 20 m^3^ per day; *T* is the time spent at the impact site per day (approximately 8 h for workers and 24 h for residents); *A*_skin_ is the skin surface area (5700 cm^2^ for adults and 2800 cm^2^ for children);^19^ ADAF is defined as an age-dependent adjustment factor (adult: 1; children: 3);^21,22^ SAF is the skin adherence factor (0.7 mg cm^−2^ per day for adult and 0.2 mg cm^−2^ per day for children);^19^ DAF is dermal absorption factor (0.001);^23^ EF is the number of days spent at the impact site annually (equal to 260 days for workers and 365 days for residents); ED is the number of years spent at the impact site in a lifetime (equal to 30 years for workers, 24 years for adult residents, and 6 years for children); BW is the body weight of an adult (70 kg); AT is the average time (70 × 365 days); CF is a conversion factor (10^−6^ kg mg^−1^); CR is the cancer risk due to metal exposure over an expected lifetime of 70 years; and SF is the cancer slope factor (kg day mg^−1^).

## Results and discussion

### Mass of particulate matter

Table 1 shows the mass concentrations of atmospheric PM of different particle sizes in the tire manufacturing plant. The concentrations of total PM in the surrounding and CB feeding areas were 107.66 ± 54.43 and 944.8 ± 456.4 μg m^−3^, respectively. The mass concentrations in the feeding area were approximately 8.8 times higher than those in the surrounding area. In the surrounding area, particles with the highest mass by size included PM_0.56–1.0_ (14.20 ± 9.11 μg m^−3^), followed by PM_0.32–0.56_ (9.64 ± 5.23 μg m^−3^) and PM_1.0–1.8_ (9.49 ± 4.86 μg m^−3^). The mass ratios of PM_0.1_ (UFPs) and PM_1_ relative to TSP were 13.84% ± 4.88% and 50.84% ± 4.29%, respectively. Overall, the mass ratios of PM_10_ relative to TSP ranged from 80.66% to 89.11% with an average of 85.36% ± 2.48%.

Concentrations (mean ± SD) of particulate matter of various sizes (unit: μg m^−3^) in the ambient air of the carbon black feeding and surrounding areas of a tire manufacturing

**Table:** 

Aerodynamic diameter (μm)	Surrounding area	CB feeding workplace
>18	8.29 ± 4.92	250.33 ± 170.02
10–18	7.77 ± 4.49	72.18 ± 36.65
5.6–10	8.72 ± 4.61	184.27 ± 108.56
3.2–5.6	9.48 ± 4.96	166.85 ± 70.80
1.8–3.2	8.91 ± 4.18	83.53 ± 36.62
1.0–1.8	9.49 ± 4.86	49.49 ± 19.08
0.56–1.0	14.20 ± 9.11	44.26 ± 24.36
0.32–0.56	9.64 ± 5.23	24.43 ± 8.88
0.18–0.32	9.25 ± 4.06	27.08 ± 9.83
0.1–0.18	7.17 ± 3.93	23.34 ± 8.03
0.056–0.1	6.28 ± 3.46	9.70 ± 5.84
<0.056	8.47 ± 5.45	9.31 ± 3.59
Total	107.66 ± 54.43	944.76 ± 456.36

**Table:** 

PM_10_ (PM_10_/TSP)	91.59 ± 45.66 (85.36 ± 2.48%)	622.24 ± 268.49 (68.10 ± 7.60%)
PM_1_ (PM_1_/TSP)	54.99 ± 28.06 (50.84 ± 4.29%)	138.11 ± 53.33 (16.91 ± 7.45%)
UFPs (UFPs/TSP)	14.74 ± 8.01 (13.84 ± 4.88%)	19.01 ± 7.02 (2.67 ± 1.92%)
MMD (μm)	0.56 ± 0.11	2.28 ± 0.61

Differences in particle mass and size distributions between the surrounding and CB feeding areas were compared. Table 1 shows that the UFPs/TSP and PM_1_/TSP ratios in the CB feeding area were 2.67% ± 1.92% and 16.91% ± 7.45%, respectively. The mass ratio of particles sized below 1 μm in the CB feeding area was significantly less than that in the surrounding area. However, the PM_1_ mass was distributed over more than 50% of the surrounding area, which suggests that the total mass of airborne particles is still incorporated with the submicron particles in this study.

### Distribution of metallic elements in different-size particles

Table 2 shows the mean concentrations of metallic elements collected from the atmosphere of the surrounding and CB feeding areas. The mean concentrations of total metals of all particle sizes were 1034.3 ± 355.8 and 13 999.6 ± 8362.4 ng m^−3^, respectively. The concentrations of total metals in the CB feeding area were approximately 13.5 times higher than those in the surrounding area. The most abundant elements in all fractions obtained from the CB feeding area were Zn (8622.0 ± 5679.0 ng m^−3^), Al (3113.3 ± 2017.1 ng m^−3^), and Fe (1519.1 ± 875.0 ng m^−3^). Zn particles made up the highest proportion (57.3% ± 10.4%) of metals relative to the total metal concentration at all particle sizes. ZnO powder is used as an activator during the tire manufacturing process;^24^ it also adds strength to rubber compounds, improves their resistance to heat and abrasion, and helps guard against ultraviolet degradation.

**Table:** Mean concentrations and weight percentages of particle-bound heavy metals in the carbon black feeding and surrounding areas

Metals	Surrounding area		CB feeding process	
	Concentration (ng m^−3^)	Weight percentage (%)	Concentration (ng m^−3^)	Weight percentage (%)
Al	224.2 ± 186.4	19.9 ± 8.6	3113.3 ± 2017.1	23.3 ± 4.6
Cd	4.8 ± 1.3	0.5 ± 0.3	30.5 ± 17.1	0.4 ± 0.3
Co	2.6 ± 1.4	0.3 ± 0.1	58.8 ± 37.5	0.8 ± 0.6
Cr	33.8 ± 5.3	3.6 ± 1.1	74.5 ± 27.1	1.0 ± 0.9
Cu	10.7 ± 7.9	0.9 ± 0.5	53.2 ± 20.9	0.7 ± 0.6
Fe	403.2 ± 137.9	39.2 ± 6.9	1519.1 ± 875.0	11.0 ± 1.2
Ga	30.9 ± 6.6	3.2 ± 1.2	94.6 ± 23.7	1.2 ± 1.0
In	7.1 ± 3.7	0.7 ± 0.3	12.6 ± 10.7	0.2 ± 0.1
Mn	24.2 ± 10.2	2.6 ± 1.3	87.0 ± 37.2	0.8 ± 0.4
Ni	18.5 ± 7.7	1.9 ± 0.9	31.5 ± 20.0	0.3 ± 0.2
Pb	50.0 ± 21.9	4.9 ± 1.2	112.1 ± 38.8	1.0 ± 0.5
Sr	20.9 ± 7.1	2.1 ± 0.9	190.3 ± 27.1	2.1 ± 1.9
Zn	203.5 ± 75.9	20.2 ± 6.9	8622.0 ± 5679.0	57.3 ± 10.4
Total	1034.3 ± 355.8		13 999.6 ± 8362.4	

The most abundant elements (Fe, Al, and Zn) in all fractions in the surrounding area were similar to those in the CB feeding area. The abundance of these three metal elements is believed to be influenced by CB feeding emissions. Fe, not Zn, was the most abundant metal relative to the total metal concentration in the surrounding area. Individual metal elements are affected to an appreciable extent by other pollution sources. Heavy-duty diesel vehicles and forklift trucks are operated near the tire factory fence, causing increases in Fe concentration in the surrounding area. Previous studies have indicated that Fe could be emitted by both exhaust (diesel emissions)^25,26^ and non-exhaust (brake wear and road dust)^24,27^ traffic sources. Diesel exhaust is the most important traffic-related nanoparticle source.^28^Table 3(a) indicates that Al, Cu, Fe, and Pb are the most abundant metallic elements in UFPs in the surrounding area. These metals (Al, Cu, and Fe) are mainly associated with diesel sources.^25,26^ To obtain further information of the main Pb source in the surrounding area, we applied partial correlation to measure the degree of association between two random variables (Fe, Pb, and Cu) with the effect of a set of controlling random variables removed (Al); the results of this analysis are shown in Table 3(b). Analysis indicated that Fe and Cu were highly correlated with each other (0.81, *p* < 0.05) and that Pb was lowly negatively correlated with Fe and Cu (−0.43 and −0.24, respectively; *p* > 0.05). These results reveal that Pb in UFPs is not likely emitted by CB feeding or traffic sources. By contrast, other metals (*e.g.*, Al, Fe, and Zn) in UFPs in the surrounding area could be associated with tire manufacturing plant emissions.

Correlation of heavy metal concentrations in UFPs in the surrounding area^a^

**Table:** 

(a) Spearman correlation													
	Al	Fe	Zn	Sr	Cu	Mn	Ni	Pb	Cd	Co	Cr	Ga	In
Al	1												
Fe	0.82**	1											
Zn			1.00										
Sr				1.00									
Cu	0.87**	0.94**			1.00								
Mn						1.00							
Ni				0.76**			1.00						
Pb	0.91**	0.65*			0.74**			1.00					
Cd				0.63*			0.68*		1.00				
Co							0.71*		0.79**	1.00			
Cr							0.62*				1.00		
Ga				0.61*			0.65*				0.64*	1.00	
In			0.85					0.70					1

a *significant level (*p* < 0.05, two-tailed); **significant level (*p* < 0.01, two-tailed).

**Table:** 

(b) Particle correlation: control variable (Al)			
	Fe	Pb	Cu
Fe	1		
Pb	−0.43	1	
Cu	0.81**	−0.24	11

**Fig. 2:**
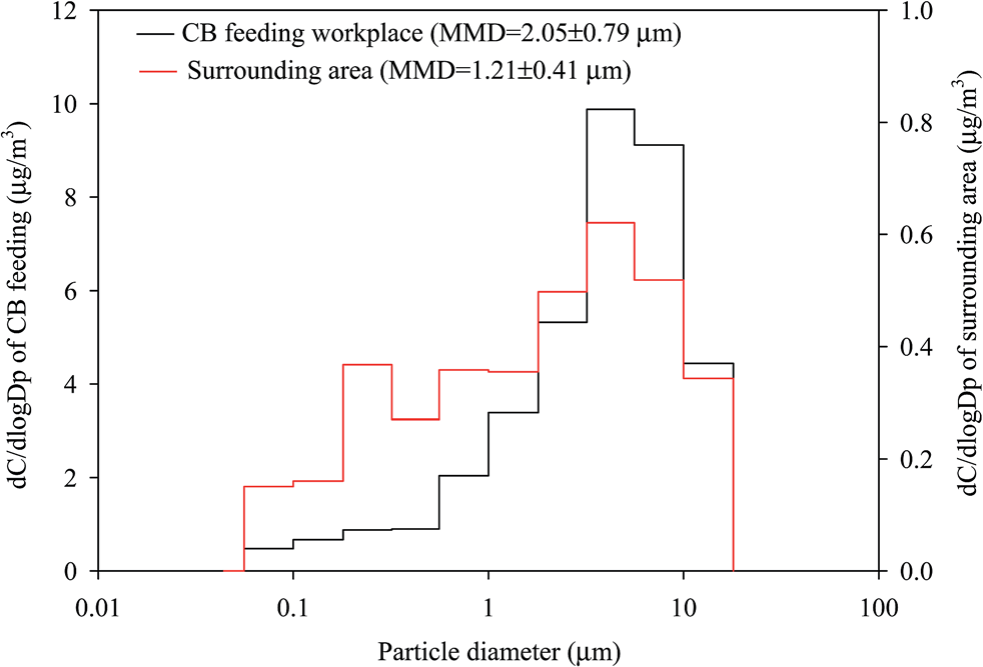
Size distributions of total particle-bound heavy metals in the ambient air of the CB feeding and surrounding areas. (a) CB feeding workplace. (b) Surrounding area.

**Fig. 3:**
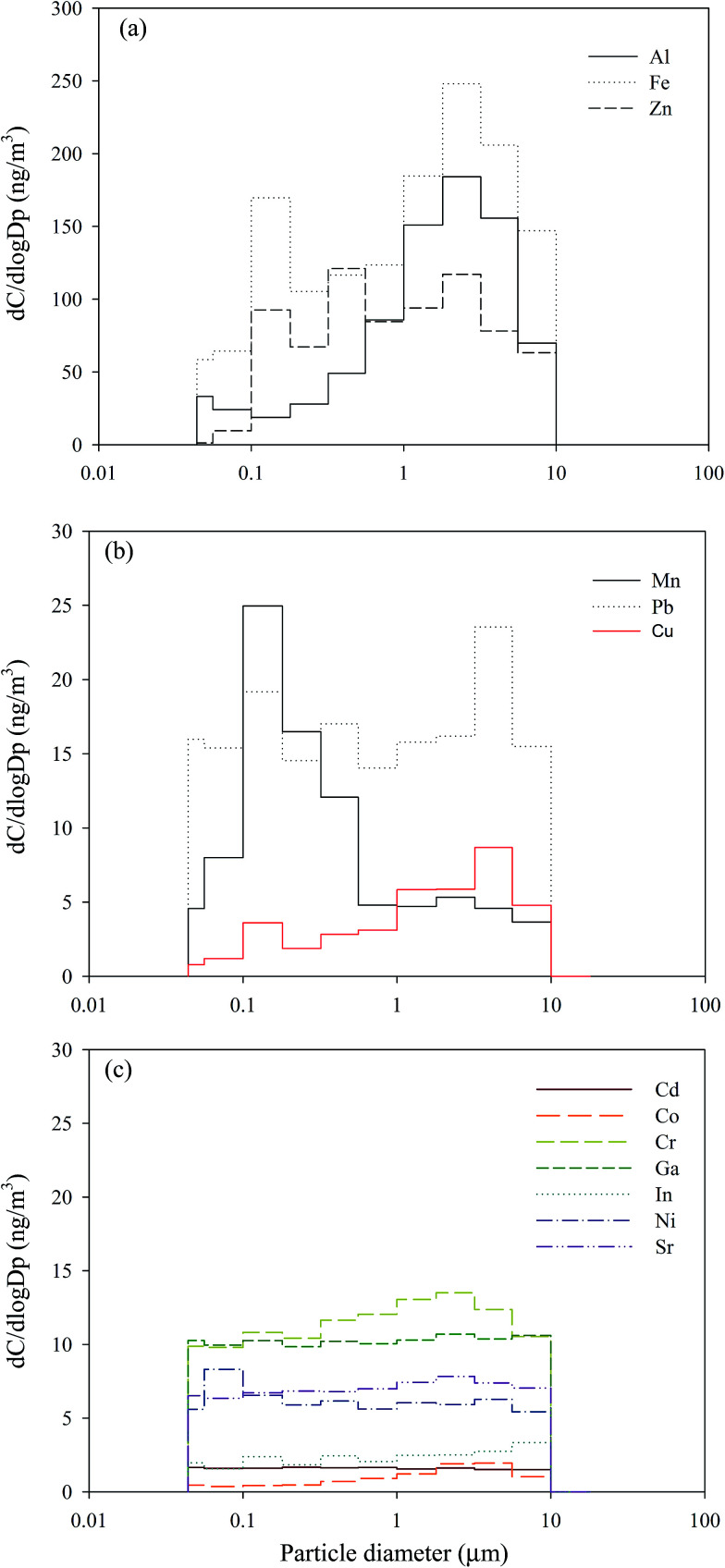
Size distributions of individual particle-bound heavy metals in the ambient air of surrounding areas.

Fig. 2 shows the mean normalized size distributions of total metal concentrations in the atmosphere of the CB feeding and surrounding areas; a unimodal distribution located at 5.6–10 μm was observed at the two sites. The mass median diameter (MMD) of the total metal concentrations in the surrounding and CB feeding areas were 1.21 ± 0.41 μm and 2.05 ± 0.79 μm, respectively, which suggests that the airborne total metals in these sites were incorporated in submicron and fine particles, respectively. Fig. 3 shows the mean normalized size distributions of individual metal concentrations in surrounding areas. Fig. 3(a) and (b) show that Al, Mn, and Cu have a unimodal distribution, exhibiting a major peak at 3.2, 0.18, and 5.6 μm, respectively. Fig. 3(a) shows that Al and Fe, exhibiting a major peak at 3.2 μm, are the most abundant metals in the surrounding area. On the contrary, Fig. 3(c) shows that the other metals do not have significant variances in particle-size distributions in the surrounding area. Although the exposure hazard of submicron-sized particles for residents was higher than that of fine particles for workers, long-term inhalation of high toxic metal levels in the CB feeding area may lead to adverse health effects among workers. Assessment of the CR presented by carcinogenic metals is discussed later in this study.

### Comparison of the sources and contents of heavy metals

**Fig. 4:**
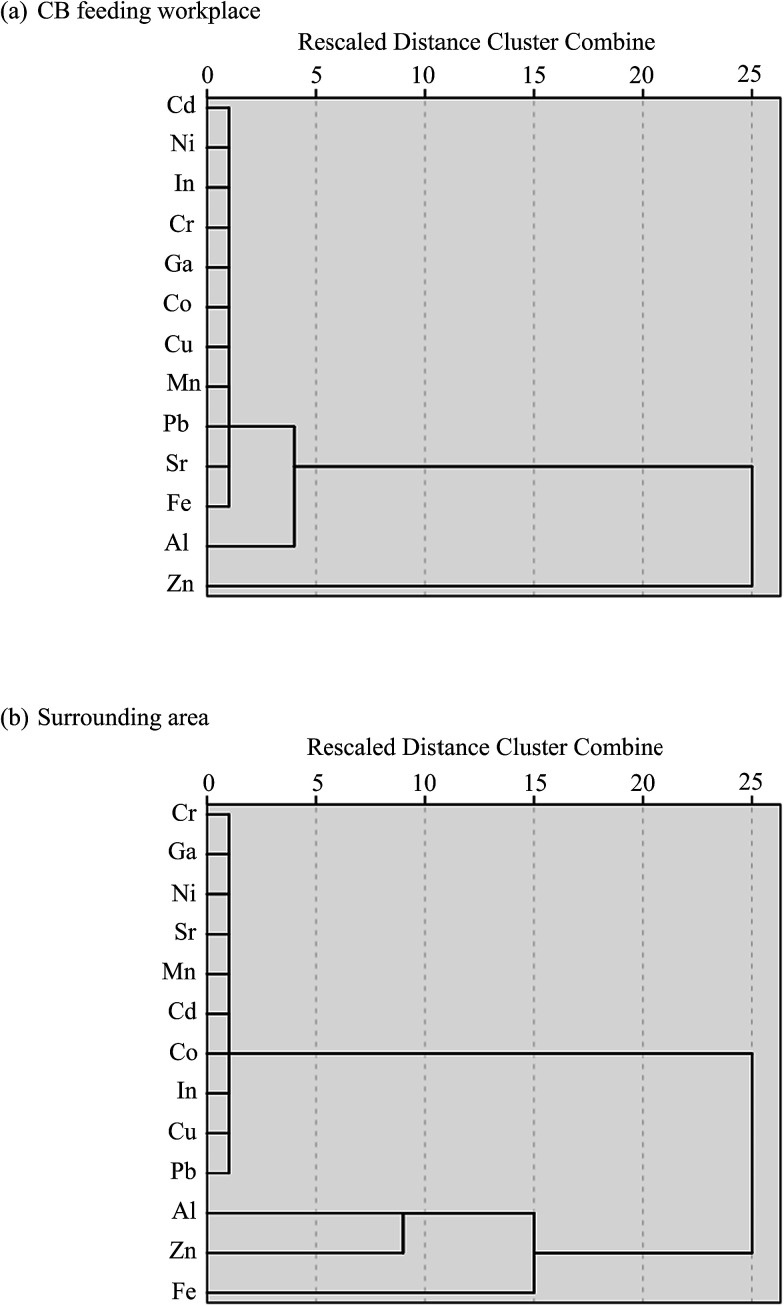
Dendrogram results of the Ward method of hierarchical cluster analysis of 13 elements in the (a) CB feeding and (b) surrounding areas.

While correlation analysis can illustrate that two metals come from the same source, it cannot group these sources accordingly. We applied cluster analysis to identify differences in emission sources by clustering samples (13 metals in all size particles) with similar heavy metal contents between the CB feeding and surrounding areas. Cluster analysis is used for exploratory data mining and can group a set of objects into more mutually exclusive unknown groups based on a combination of internal variables. Most research uses hierarchical clustering according to Ward's method to achieve this goal.^29–31^Fig. 4 shows the dendrogram results of hierarchical cluster analysis of 13 metals in all particle sizes in the CB feeding and surrounding areas. Prior to cluster analysis, the variables were standardized by means of *z*-scores; thereafter, the Euclidean distances of similarities in the variables were calculated. Fig. 4(a) presents two large subgroups in the CB feeding area: the first group contains only the variable Zn while the second one includes Al and other metals. The Zn in the first group is mainly obtained from the addition of ZnO powder in the CB feeding process, and the metals in the second group are related to raw CB. To identify the original source of metals in the surrounding area, two large subgroups were determined: the first group contained only the variables Al, Fe, and Zn and the second group included Pb and other metals (Fig. 4(b)). In this case, the first group is related to emissions from the CB feeding process. Unfortunately, the probable sources of the second group were difficult to identify because the metal contents in this group did not show significant variances. The characteristics of metal-containing particles are affected by their emission sources. Therefore, the contributions of metallic contents in different particle sizes could be used as a marker of probable exhaust sources.

**Fig. 5:**
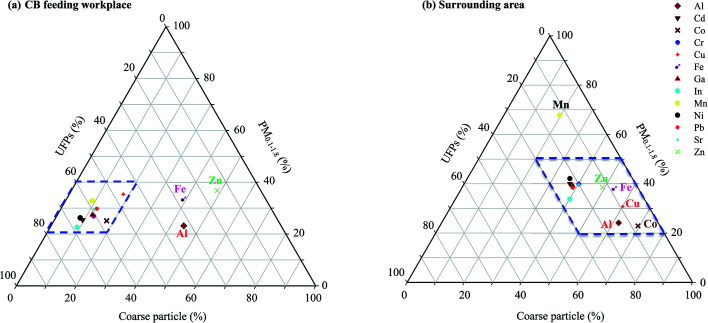
Triangular diagrams showing heavy metal speciation in different particle sizes: (a) carbon black feeding and (b) surrounding areas.

Fig. 5(a) and (b) respectively illustrate the total relative contents of metals in particles sampled at the CB feeding and surrounding areas. Fig. 5(a) shows that the Al, Fe, and Zn metal contents in coarse particles (49.1–62.0%) contributed the most to the total PM content, followed by those in PM_0.1–1.8_ (23.2–36.8%) and UFPs (14.5–32.5%). Other metal contents were distributed mainly in UFPs (46.3–67.7%), thus demonstrating that UFPs pose greater adverse health effects than larger particles per unit mass in the CB feeding area. Since no other raw materials were fed in the workshop, the metal contents of the collected particles clearly represent the characteristics of raw CB nanoparticles.

Fig. 5(b) shows that the mean percentage contributions of Al, Fe, Zn, Cu, and Co in the coarse particles ranged from 49.1% to 69.1%, thus indicating that the content contributions of Al, Fe, and Zn to coarse particles were affected by CB feeding area emissions. Although Cu and Co may be emitted by crustal elements, the results of the present study suggest that most of the Cu in the coarse particles comes from the road dust of the surrounding area. Road dust contains dust from the brakes and tires of vehicles. The average mass median diameters of brake dust range from 0.62 μm to 2.49 μm.^32^ The Cu content of brake dust has been recognized to be a significant pollutant,^24,33^ contributing 47% of the total Cu load in urban runoff.^24,34^ Cu is used to control heat transport.^35^ A large amount of Mn, which probably comes from the fumes produced during manual metal arc welding in the tire plant, was observed in PM_0.1–1.8_ (67.8%). The diameters of most of the particles found in emitted welding fumes range from 0.56 μm to 0.1 μm.^36^ Besides Fe, Cr, and Ni, welding fumes contain high concentrations of Mn and Zn.^37^ Some combinations of multiple elements have been found to be specific to particular sources or process, thereby enabling these particle classes to be used as markers.^38^ However, most of the toxic metals found in PM_0.1–1.8_ were observed in the surrounding atmosphere of the tire manufacturing plant.

### Deposition fluxes in the respiratory system and health-risk assessment

**Fig. 6:**
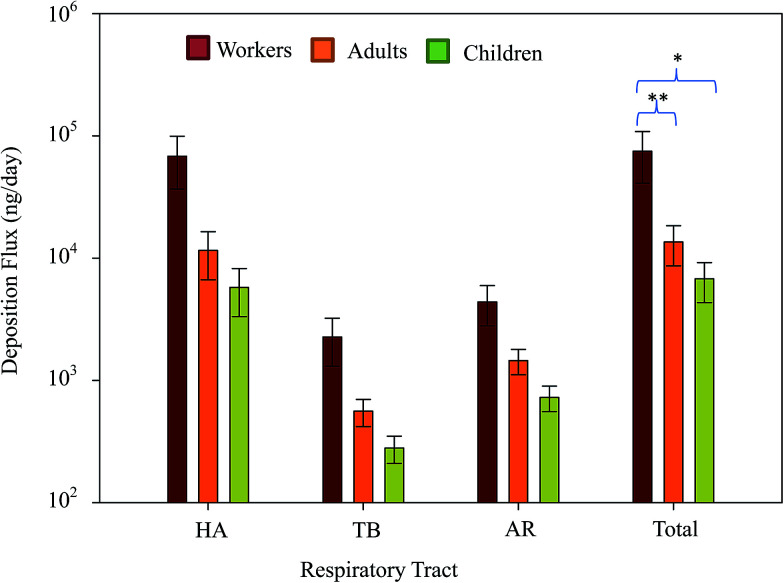
Deposition fluxes and 95% confidence intervals of total particle-bound heavy metals in the human respiratory tracts of workers, adults, and children. *p* < 0.05 and **p < 0.01 compared to the workers.

Fig. 6 presents the deposition fluxes of the means and 95% confidence intervals (CI) of total particle-bound heavy metals in the human respiratory tracts of workers, adults, and children. The mean total deposition fluxes of atmospheric particle-bound heavy metals in the HA, TB, and AR were 68 081.5 (CI: 36 817.5–99 345.5), 2268.0 (CI: 1307.4–3228.5), and 4374.1 (CI: 2788.9–5959.4) ng per day, respectively. The total deposition fluxes (HA + TB + AR) of atmospheric particle-bound heavy metals in adults and children were 13 537.7 and 6768.9 ng per day, respectively. Mean values following the sequence HA > AR > TB demonstrated similar trends among workers, adults, and children. The ratios of total mean deposition fluxes in workers/adults, workers/children, and adults/children were approximately 5.5, 11.0, and 2.0, respectively. Fig. 6 shows the differences in the total deposition fluxes of particle-bound heavy metals among workers, adults, and children, which were determined using Kruskal–Wallis test. As the results showed very strong evidence of a difference (*p* < 0.001) between the mean ranks of at least one pair of groups, the Dunn's pairwise tests were carried out for three pairs of groups. Differences were observed between workers–adults and workers–children (*p* < 0.05 and *p* < 0.001, respectively, adjusted using the Bonferroni correction). This study compared the mean total deposition fluxes of adults and children with those in an e-waste recycling zone,^18^ and the total deposition fluxes of metals in adults and children near the tire plant were about 0.4 times lower than those in the recycling zone. This result reveals that the enclosed plant control management style implemented in the CB feeding area was is better than open treatment method practiced in the e-waste recycling zone. Thus, enclosed plant control management could reduce the adverse health effects of tire manufacturing on residents living near the tire plant.

**Fig. 7:**
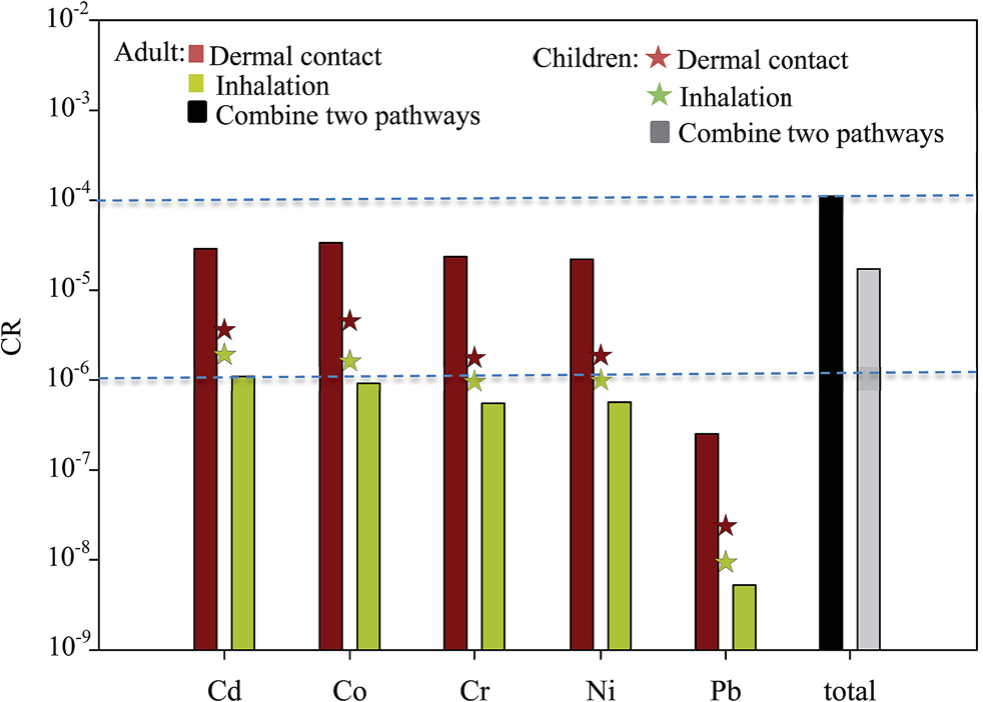
Cancer risk assessment of carcinogenic metals *via* dermal contact and inhalation.

To assess the health risk posed by toxic metals in the ambient air of surrounding areas to residents (both adults and children), the carcinogenic effects of toxic metals *via* the major exposure pathways of inhalation and dermal contact were estimated. The CRs of toxic metals in the total PM presented by these two exposure pathways are shown in Fig. 7. The dermal contact CR of the ambient air of the surrounding area was highest for Co (3.3 × 10^−5^), followed by Cd (2.9 × 10^−5^) and Cr (2.3 × 10^−5^) for adults. The mean dermal contact CR of five elements for adults was approximately 10 times higher than that for children. Means inhalation of the CR presented by Cd, Co, Cr, Ni, and Pb as determined by the ICRP model. Among the CRs determined, the CR presented by Cd inhalation of the total PM for adults and children was the highest, followed by those of Co and Ni. Because the inhalation rates of the total PM in the respiratory tract were not 100%, the CR presented by dermal contact was higher than that presented by inhalation. These results are consistent with those of other studies.^39–43^ The CRs presented by inhalation for children were higher than those for adults. The CR of Cd inhalation for adults and children and that of Co inhalation for children exceeded the 10^−6^ benchmark level. The integrated risks presented by five elements *via* two exposure pathways to adults and children were 1.1 × 10^−4^ and 1.7 × 10^−5^, respectively, thus demonstrating the high cumulative CR posed by these metals to local residents (both adults and children).

Toxic metals have attracted growing attention in urban cities near industrial plants because of the effects of PM on public health. The daily intakes of toxic constituents *via* ingestion, dermal contact, and inhalation have been calculated, and, among the three pathways, the respiratory system was found to provide the easiest exposure route. The adverse effects of inhaled toxic metals can be widely found in previous publications that used 75% penetration levels to assess fractions containing high concentrations of total particle-bound contaminants penetrating the lungs.^16,44–46^ In this study, the majority (59.02% ± 35.58%) of the deposition amounts among residents was observed in the HA; only 2.68% ± 0.32% and 7.02% ± 0.49% of the total particles were deposited in the TB and AR, respectively (Table 4). The mean total portions deposited in the respiratory tract of residents and workers were 88.24% and 82.56%, respectively. A similar trend was observed in the e-waste recycling zone. The results of the present study suggest that the inhalable fraction deposited into the AR was much lower than the empirical value of 75%. Therefore, assessments based on total contaminants could show overestimated exposure risks.

**Table:** Mean deposited fractions of total particle-bound metals in the human respiratory tract

Site	HA	TB	AR
Surrounding area	59.02 ± 35.58	2.68 ± 0.32	7.02 ± 0.49
Workplace	73.22 ± 2.59	2.49 ± 0.29	5.19 ± 1.13

The ICRP model used in the current study presents a number of limitations, such as underestimation of the deposition of ultrafine particles in the lower human respiratory tract^47^ and ignorance of the presence and commensurate effects of naturally occurring structural elements of the lungs (*e.g.*, cartilaginous rings, carinal ridges), which have been demonstrated to affect the motion of inhaled air.^48^ While these limitations could affect the assessment of CRs, the results still provide important insights into novel protection strategies associated with exposure to particle-bound metals.

## Conclusions

This study was conducted to investigate the effects on human health and size distributions of CB particles in a tire manufacturing plant. In the surrounding area, particles with the highest mass by size were PM_0.56–1.0_, and the MMD of the total airborne mass was mainly incorporated in submicron particles (PM_1_). The most abundant elements (Fe, Al, and Zn) in all fractions of the surrounding area were similar to those of the CB feeding area. The MMDs of the total metal concentration in the surrounding and CB feeding areas were 1.21 ± 0.41 and 2.05 ± 0.79 μm, respectively. Cluster analysis revealed that the CB feeding process is the main emitter of metals in the surrounding area. The mean values of total particle-bound heavy metals in the human respiratory tract following the sequence HA > AR > TB demonstrated similar trends among workers and residents. CR values of inhalation for children were higher than those for adults. The inhalation CRs of Cd for adults and children and Co for children exceeded the 10^−6^ benchmark level. Although the existing plant control management of tire manufacturing reduced the adverse health effects of CB on residents living near the tire plant, this study suggests that CB emission can be effectively controlled by applying negative pressure using local exhaust ventilations. Then, the CB particles can be reused by collecting them using a bag house or other equipment. The results provide new information regarding the emissions of CB manufacturing plants.

## Conflicts of interest

There are no conflicts of interest to declare.

## Supplementary Material
